# Urinary sediment microRNAs can be used as potential noninvasive biomarkers for diagnosis, reflecting the severity and prognosis of diabetic nephropathy

**DOI:** 10.1038/s41387-021-00166-z

**Published:** 2021-06-30

**Authors:** Qiuxia Han, Youcai Zhang, Tingting Jiao, Qi Li, Xiaonan Ding, Dong Zhang, Guangyan Cai, Hanyu Zhu

**Affiliations:** 1 School of Medicine, Nankai University, State Key Laboratory of Kidney Diseases, National Clinical Research Center of Kidney Diseases, First Medical Center of Chinese PLA General Hospital, Tianjin, China; 2 Department of Nephrology, First Medical Center of Chinese People’s Liberation Army General Hospital, Nephrology Institute of the Chinese People’s Liberation Army, Beijing Key Laboratory of Kidney Disease Research, Beijing, China; 3 Department of Nephrology, Jiaozuo People’s Hospital, Jiaozuo, China

**Keywords:** Biological techniques, Risk factors

## Abstract

**Background:**

Patients with both diabetes mellitus (DM) and kidney disease could have diabetic nephropathy (DN) or non-diabetic renal disease (NDRD). IgA nephropathy (IgAN) and membranous nephropathy (MN) are the major types of NDRD. No ideal noninvasive diagnostic model exists for differentiating them. Our study sought to construct diagnostic models for these diseases and to identify noninvasive biomarkers that can reflect the severity and prognosis of DN.

**Methods:**

The diagnostic models were constructed using logistic regression analysis and were validated in an external cohort by receiver operating characteristic curve analysis method. The associations between these microRNAs and disease severity and prognosis were explored using Pearson correlation analysis, Cox regression, Kaplan–Meier survival curves, and log-rank tests.

**Results:**

Our diagnostic models showed that miR-95-3p, miR-185-5p, miR-1246, and miR-631 could serve as simple and noninvasive tools to distinguish patients with DM, DN, DM with IgAN, and DM with MN. The areas under the curve of the diagnostic models for the four diseases were 0.995, 0.863, 0.859, and 0.792, respectively. The miR-95-3p level was positively correlated with the estimated glomerular filtration rate (*p* < 0.001) but was negatively correlated with serum creatinine (*p* < 0.01), classes of glomerular lesions (*p* < 0.05), and scores of interstitial and vascular lesions (*p* < 0.05). However, the miR-631 level was positively correlated with proteinuria (*p* < 0.001). A low miR-95-3p level and a high miR-631 level increased the risk of progression to end-stage renal disease (*p* = 0.002, *p* = 0.011).

**Conclusions:**

These four microRNAs could be noninvasive tools for distinguishing patients with DN and NDRD. The levels of miR-95-3p and miR-631 could reflect the severity and prognosis of DN.

## Introduction

The incidence of diabetes mellitus (DM) has gradually increased with the development of a social economy, and the number of patients with diabetes is expected to exceed 693 million by 2045 [1]. Approximately 40% of diabetic patients are prone to the development of diabetic nephropathy (DN) [2–4]. DN is one of the most common microvascular complications of diabetes and is considered the most common cause of end-stage renal disease (ESRD) [5, 6]. Many scholars have found that some patients with diabetes and kidney disease have different clinical manifestations and sensitivities to treatment than those with typical DN [7, 8]. Apart from DN, diabetic patients with kidney disease may also have non-diabetic renal disease (NDRD) [9, 10]. According to reports, the incidence of NDRD is ~53%, which varies among countries, possibly due to race, region, and selection criteria for renal biopsy [10, 11]. IgA nephropathy (IgAN) and membranous nephropathy (MN) are the major types of NDRD [11, 12]. Li et al. [13] conducted a 5-year longitudinal follow-up survival analysis and found that the prognosis of the NDRD group was better than that of the DN group, which indicates that patients with type 2 diabetes who were diagnosed with NDRD at the early stage can achieve an improved prognosis after appropriate and timely treatment measures are given. For diabetic patients with kidney disease, it is not feasible to estimate a diagnosis of DN based on experience [9, 14]. Instead, consideration should be given to diabetic patients with other kidney diseases, especially MN and IgAN, which are very common [15]. Since DN and NDRD are not the same type of disease, their pathological characteristics, clinical manifestations, treatment responses, disease progression rates, and prognoses all differ, and thus, an accurate differential diagnosis is particularly important [16]. The gold standard for distinguishing DN and NDRD is renal biopsy, but it cannot be performed routinely because of its invasiveness, high cost, and high technical requirements. Therefore, a simple, stable, and reliable identification method is an urgent clinical need. However, an ideal noninvasive diagnostic model for DN has not yet been established nationally or abroad.

MicroRNA is an endogenous non-coding single-stranded RNA consisting of 21–25 nucleotides [17]. MicroRNAs can induce the degradation of messenger RNAs and block protein translation. They also play a very important regulatory role in the occurrence and development of kidney disease [17]. Therefore, the discovery of blood- and urine-specific microRNAs in patients with DN has provided a new direction for identifying diagnostic biomarkers for DN and has enabled the timely screening and disease monitoring of DN [18]. Many studies have shown that the urine of patients with DN contains numerous exfoliated podocytes, the number of which is proportional to the severity of proteinuria [19]. Podocyte shedding is an important pathological feature of DN, and proteinuria is an important clinical feature of DN [20]. Given that urinary sediment cells are mainly derived from the kidney, detection of biomarkers from the urinary sediment of patients can more accurately capture the specific biological information changes that occur in kidney disease [21]. In addition, urinary sediment is obtained by the centrifugation of urine, this noninvasive and easy method can contribute to early screening and disease monitoring [22].

Although some studies have focused on biomarkers in blood and urine supernatants from patients with DN, no studies have investigated the role of microRNAs in the diagnosis of DN based on urinary sediment. Compared with blood sample collection, urine sample collection is more convenient, less invasive, and painless. Urinary sediment has a higher nucleic acid content and is relatively less affected by humoral metabolic factors than urine supernatant [23]. Therefore, urinary sediment is an ideal specimen for identifying noninvasive diagnostic biomarkers.

The purpose of this study was to investigate microRNA alterations among DM, DN, and NDRD and construct diagnostic models for these diseases, and to identify noninvasive biomarkers that can reflect the severity and prognosis of DN.

## Materials and methods

### Study design and subject enrollment

**Fig. 1:**
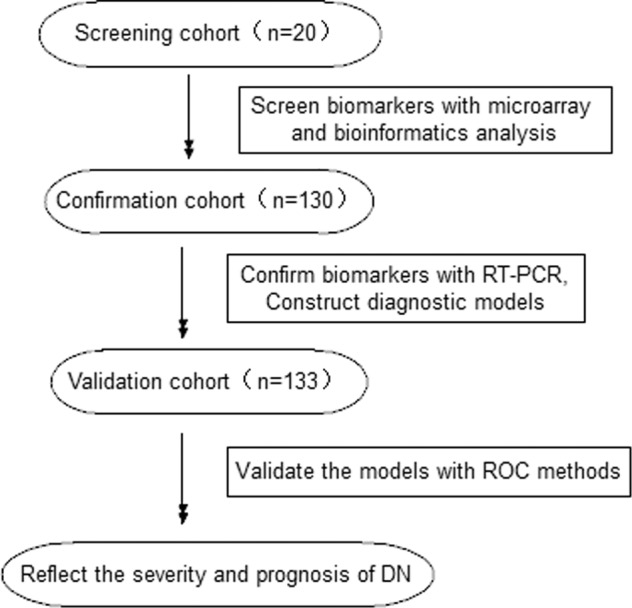
The design scheme for constructing and identifying the diagnostic models in this study. PCR polymerase chain reaction, ROC receiver operating characteristic, DN diabetic nephropathy.

In all, 283 eligible subjects at the General Hospital of the Chinese People’s Liberation Army were enrolled from January 2017 to September 2019. The study design scheme is illustrated in Fig. 1. This study was conducted in accordance with the Declaration of Helsinki for research involving human subjects and was approved by the Ethics Committee of the Chinese PLA General Hospital (No. S2014-012-01). Written informed consent for inclusion was obtained from each participant.

A diagnosis of type 2 diabetes was defined according to either the fasting plasma glucose (FPG) level or 2-h plasma glucose level after a 75 g oral glucose tolerance test (OGTT) or according to the hemoglobin A1c (HbA1c) based on the American Diabetes Association (ADA) criteria [24]. The pathological classification of DN was performed according to the Renal Pathology Society (RPS) classification system [25]. Indications for renal biopsy were matched with the criteria for NDRD in the 2007 Kidney Disease Outcomes Quality Initiative (KDOQI) guidelines [26]. As for the sample size calculation, when we set the confidence level as 0.95, the power is 0.8, the distance from mean to limits is 0.3, standard deviation is 0.4, the interval type is two-sided, the required sample size is 17.

The inclusion criteria were as follows: diagnosis of type 2 diabetes; pathological diagnosis of DN or NDRD; age over 18 years; underwent renal biopsy at our hospital; agreed to join this experimental study and voluntarily signed an informed consent form. The exclusion criteria were as follows: patients with an incomplete or unclear medical history; combined urinary tract infection, immune system disease, malignant tumor or pregnancy; a confirmed diagnosis of renal disease before a type 2 diabetes diagnosis; a clinically confirmed diagnosis of NDRD, including lupus nephritis and Henoch-Schönlein purpura nephritis; and history of familial hereditary nephropathies. To evaluate whether microRNAs in urinary sediment may be potential biomarkers that can differentiate DN from NDRD, patients with other kidney diseases accompanied by DN or NDRD and those with a pathological diagnosis of DN combined with NDRD were excluded from this study. The kidney tissues were obtained by percutaneous renal biopsy. Sections were routinely stained by hematoxylin and eosin (H&E), periodic acid–Schiff (PAS), and periodic acid silver methenamine (PASM). The pathological findings were independently evaluated by two authoritative pathologists.

### Urine collection and RNA extraction

Morning urine specimens of 150–250 ml were collected from DN and NDRD patients before renal biopsy and from DM patients and were immediately centrifuged at 3000 × *g* at 4 °C for 30 min and at 13,000 × *g* at 4 °C for 5 min. Then, the supernatant was discarded, and urinary sediment was retained. TRIzol (Invitrogen, USA) was used to extract total RNA from urinary sediment according to the manufacturer’s protocol. The concentration and purity of the total RNA were measured with a NanoDrop 2000 spectrophotometer (Thermo Scientific, USA).

### MicroRNA microarray analysis

Human microRNA microarrays from Agilent Technologies (8*60 K) containing probes for 2549 human microRNAs from the miRBase V21.0 database were used. The microarray image information was converted into spot intensity values using Scanner Control Software Rev. 7.0 (Agilent Technologies, Santa Clara, CA). Raw data were normalized by Quantile algorithm, included in the R package AgiMicroRna [27]. The microarray experiments were performed at Shanghai Biotechnology Corporation and microRNAs with altered expression levels were screened in each experimental group according to the manufacturer’s protocol. The screening cohort included 5 type 2 DM patients, 6 DN patients, and 9 NDRD (4 IgAN and 5 MN) patients.

### Reverse transcription and quantitative real-time PCR

Total RNA (800 ng) was used to perform cDNA synthesis using the miRcute plus microRNA First-strand cDNA Synthesis Kit (catalog number KR211-02; Tiangen Biotechnology Co., Ltd., Beijing) according to the manufacturer’s instructions. PCR on the samples was performed using a miRcute plus microRNA qPCR Detection Kit (catalog number FP401; Tiangen Biotechnology Co., Ltd., Beijing) and an Applied Biosystems 7500 Real-Time PCR System (Applied Biosystems, USA) according to the manufacturer’s instructions. All PCR experiments were performed in triplicate and were followed by melting curve analysis to verify specificity and identity of the qPCR products. All primers were purchased from Tiangen Biotechnology Company (Beijing); U6 served as the endogenous reference control, and the relative microRNA expression levels were expressed as 2^−ΔΔCT^.

### Statistical methods

Normally distributed data were expressed as the mean± standard deviation (SD) and were compared using unpaired Student’s *t*-tests. Nonnormally distributed data were expressed as medians with the corresponding 25th and 75th percentiles (interquartile range) and were compared using Mann–Whitney U-tests. All tests are two-sided. Values of the relative microRNA expression levels that were out of the average background ± 2 standard deviations (SDs) were removed from each data point to minimize possible systematic variation. Four diagnostic models of type 2 DM (Model DM), DN (Model DN), NDRD-IgAN (Model IgAN), and NDRD-MN (Model MN) were constructed according to the relative microRNA expression levels based on a forward stepwise logistic regression analysis. The diagnostic performance of the diagnostic models was evaluated by receiver operating characteristic (ROC) curve analysis. Pearson correlation was used to test associations between relative microRNA expression levels and clinical and pathological parameters related to the severity of DN. Cox regression, Kaplan–Meier survival curves, and the log-rank test were performed to analyze dialysis-free survival. *P* values <0.05 were considered statistically significant. Statistical analyses were performed using SPSS statistics 21.0 software (version 21.0 SPSS, Chicago, IL, USA) and GraphPad Prism software (Vision 8, San Diego, CA, USA).

## Results

### MicroRNA microarray analysis in the screening phase

MicroRNA microarrays were used to analyze the global expression profiles of the four groups (DM, DN, IgAN, and MN). The microRNA profiling of global urinary sediment from patients in the different groups is shown in Supplementary Fig. S1. The volcano plots of microRNAs evaluated in the screening phase are reported in Supplementary Figs. S2–S7. We selected microRNAs with fold changes >2 or <0.5 and *p* values <0.05. The total numbers of microRNAs evaluated in the screening phase are reported in Supplementary Tab. S1. We then analyzed the data combined with the target gene prediction websites and biological information analysis. Finally, the sample size was expanded for verification.

### Confirmation of the candidate microRNAs and construction of the diagnostic models

In all, 130 participants were enrolled in the confirmation cohort. The baseline characteristics of the subjects are shown in Supplementary Tab. S2. A comparison of urinary sediment microRNA expression profiles among the different groups in the confirmation cohort is shown in Table 1. The expression levels of four microRNAs were different in the DN group versus all other groups. The relative expression levels of miR-95-3p and miR-631 were increased in the DN group compared with the DM group (both *p* < 0.001), IgAN group (*p* < 0.05 and *p* < 0.01), and MN group (*p* < 0.001 and *p* < 0.01). The miR-185-5p level was increased in the DN group compared with the DM group (*p* < 0.001) but was decreased compared with the IgAN group (*p* < 0.01) and MN group (*p* < 0.05). The miR-1246 level was increased in the DN group compared with the DM group (*p* < 0.001) and the MN group (*p* < 0.01) but was decreased compared with the IgAN group (*p* < 0.05).

**Table 1:** Comparison of urinary sediment microRNA expression profiles among different groups in the confirmation cohort.

	miR-95-3p	miR-185-5p	miR-1246	miR-631
DM Group	0.246 ± 0.141***	0.467 ± 0.178***	0.421 ± 0.161***	0.527 ± 0.208***
DN Group	0.814 ± 0.380	0.828 ± 0.203	0.891 ± 0.217	0.843 ± 0.243
IgAN Group	0.671 ± 0.169*	1.051 ± 0.269**	1.017 ± 0.283*	0.728 ± 0.178**
MN Group	0.375 ± 0.119***	0.953 ± 0.278*	0.721 ± 0.201**	0.608 ± 0.174**

*DM* diabetes mellitus (type 2), *DN* diabetic nephropathy, *IgAN* IgA nephropathy, *MN* membranous nephropathy.

*Other groups compared with DN group, *p < 0.05; **p < 0.01; and ***p < 0.001.

We then used logistics regression to establish a diagnostic model for DM, DN, IgAN, and MN based on the microRNA expression level in the confirmation cohort.

The DM diagnostic model is as follows:

Model DM = 1/1 + e^−(21.536−19.775×miR-185-5p −15.082×miR-1246)^

The DN diagnostic model is as follows:

Model DN = 1/1 + e^−(−5.507+3.973×miR-95-3p+3.773×miR-631)^

The IgAN diagnostic model is as follows:

Model IgAN = 1/1 + e^−(−8.031+3.453×miR-185-5p+3.846×miR-1246)^

The MN diagnostic model is as follows:

Model MN = 1/1 + e^−(−2.328-2.297×miR-95-3p+4.083×miR-185-5p −2.279×miR-1246)^

**Fig. 2:**
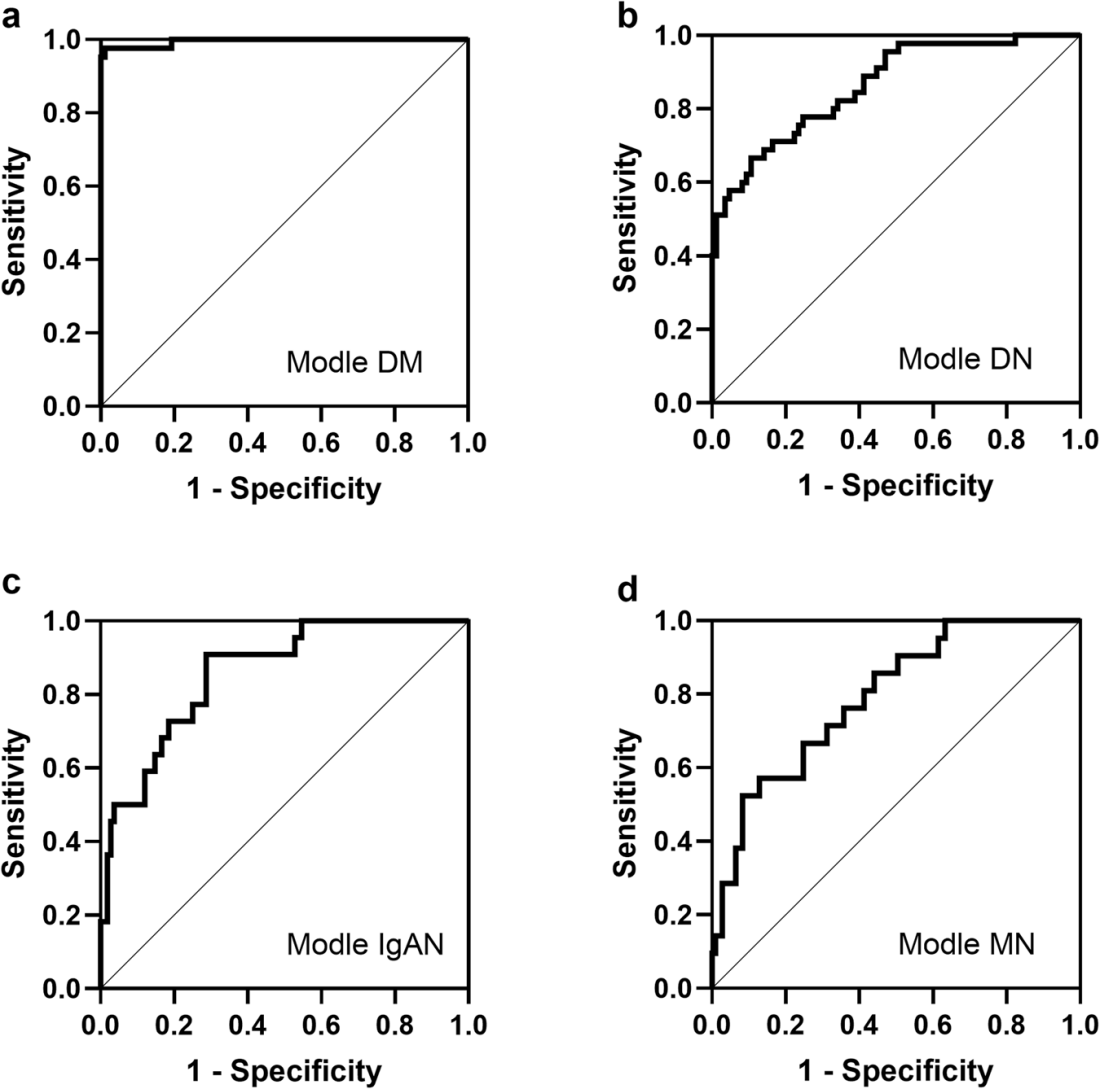
The diagnostic accuracy of the diagnostic models was determined by ROC analysis in the confirmation cohort. **a**–**d** The ROC analysis is shown for Model DM, Model DN, Model IgAN, and Model MN in the confirmation cohort. DM diabetes mellitus, DN diabetic nephropathy, IgAN IgA nephropathy, MN membranous nephropathy.

Evaluation parameters of the diagnostic model are shown in Table 2. The accuracies of our four models are 0.910, 0.865, 0.887, and 0.872. These parameters showed that our model is reliable. In addition, we tested the diagnostic model using the ROC method based on the results (predicted probability) of logistic regression (Fig. 2). The areas under the curve of these four models are 0.995, 0.863, 0.859, and 0.792. These data further show that the diagnostic model is reliable (Supplementary Tab. S3).

**Table 2:** Evaluation parameters of the diagnostic model based on the logistics regression results.

Model	Positive predictive value	Negative predictive value	Accuracy
Model DM	0.804	0.976	0.910
Model DN	0.725	0.951	0.865
Model IgA	0.778	0.904	0.887
Model MN	0.818	0.877	0.872

*DM* diabetes mellitus, *DN* diabetic nephropathy, *IgAN* IgA nephropathy, *MN* membranous nephropathy.

### Validation of the diagnostic models

**Fig. 3:**
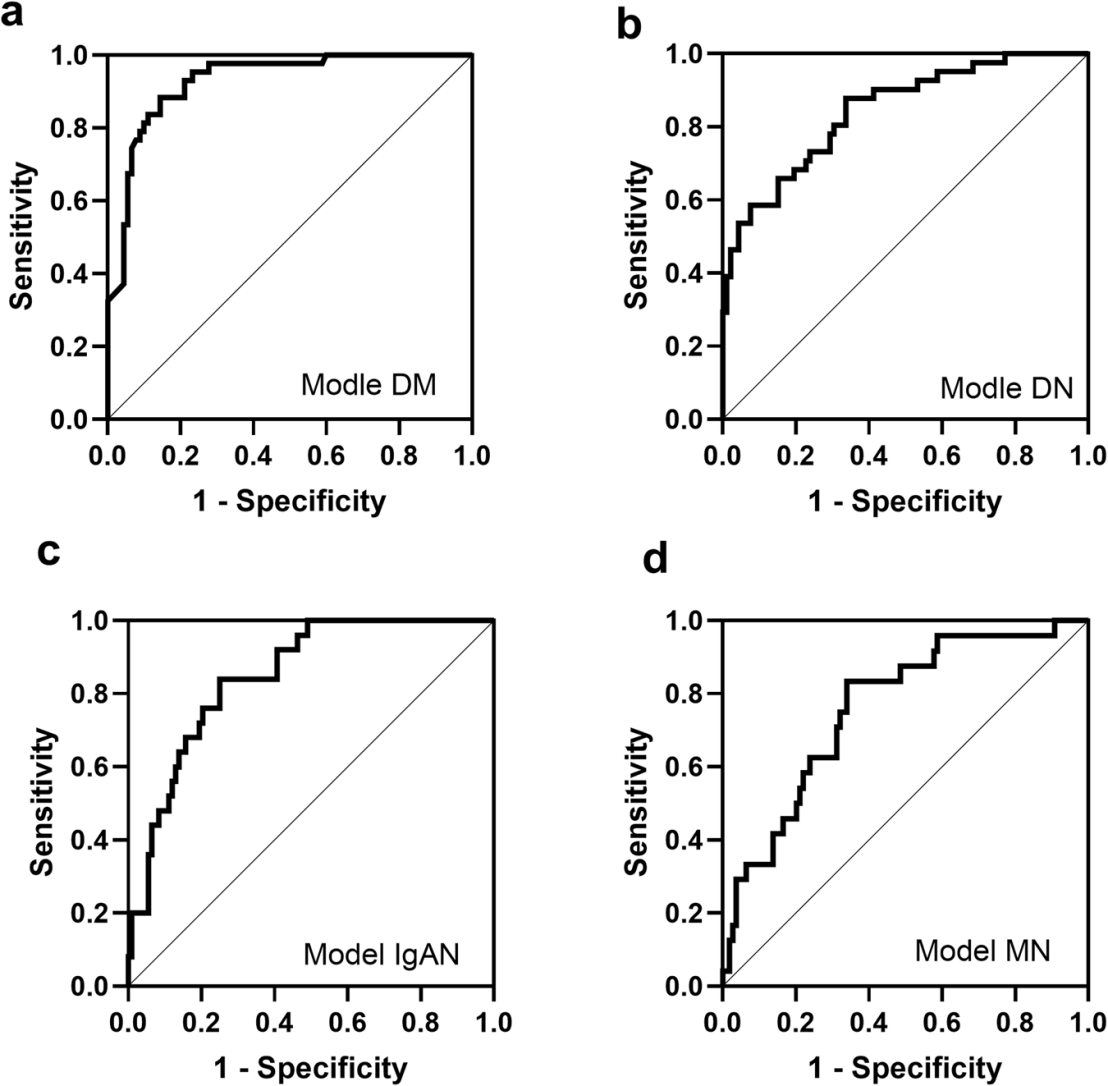
The diagnostic accuracy of the diagnostic models was determined by ROC analysis in the validation cohort. **a**–**d** The ROC analysis is shown for Model DM, Model DN, Model IgAN, and Model MN in the validation cohort. DM diabetes mellitus, DN diabetic nephropathy, IgAN IgA nephropathy, MN membranous nephropathy.

The constructed models based on the confirmation cohort were then applied to the validation cohort of 133 patients to evaluate their diagnostic power. As shown in Fig. 3, all candidate microRNAs were generally coincident with the results in the confirmation cohort. The details of the ROC analyses of the constructed models are shown in Supplementary Tab. S3.

The ROC curves indicated that the Model DM (AUC = 0.928) had the highest diagnostic accuracy for distinguishing DM from the other three diseases (Fig. 3a). The ROC curves indicated that the Model DN (AUC = 0.844) had high diagnostic accuracy for distinguishing DN from the other three diseases (Fig. 3b). Moreover, the ROC curves indicated that the Model IgAN (AUC = 0.849) had high diagnostic power for distinguishing IgAN from the other three diseases (Fig. 3c). The ROC curves indicated that the Model MN (AUC = 0.761) had moderate diagnostic power for distinguishing MN from the other three diseases (Fig. 3d).

### Correlations between relative microRNA expression levels and severity of DN

To investigate the correlations between relative microRNA expression levels and the severity of DN, we performed a Pearson correlation analysis of microRNA expression levels and clinical and pathological parameters related to the severity of DN (Table 3). The level of miR-95-3p was positively correlated with the estimated glomerular filtration rate (eGFR) (*p* < 0.001) but was negatively correlated with serum creatinine (*p* < 0.01), classes of glomerular lesions (*p* < 0.05), and scores of interstitial and vascular lesions (*p* < 0.05). The level of miR-631 was positively correlated with proteinuria (*p* < 0.001), whereas the levels of miR-185-5p and miR-1246 showed no correlation with any clinical and pathological parameters related to the severity of DN.

**Table 3:** Pearson correlations of relative microRNA expression levels and clinical parameters related to the severity of diabetic nephropathy.

	miR-95-3p	miR-185-5p	miR-1246	miR-631
Proteinuria (g/24 h)	−0.184	0.084	0.055	0.532***
eGFR (ml/min/1.73 m^2^)	0.429***	0.039	0.083	0.078
Serum creatinine	−0.369**	−0.161	−0.062	−0.143
Classes of glomerular lesions	−0.280*	−0.096	0.001	−0.115
Scores of Interstitial and vascular lesions	−0.297*	−0.053	−0.187	−0.002

The estimated glomerular filtration rate (eGFR) was calculated using the Chronic Kidney Disease Epidemiology Collaboration (CKD-EPI) equation.

*p < 0.05; **p < 0.01; and ***p < 0.001.

### Association of microRNAs and dialysis-free survival of DN patients

**Fig. 4:**
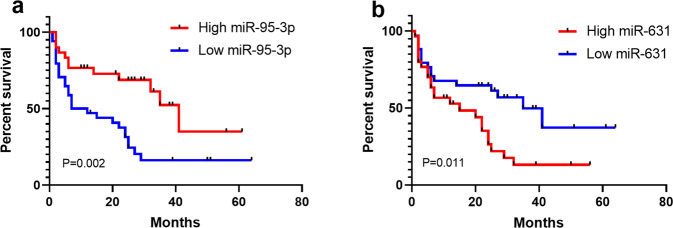
Kaplan–Meier analysis of dialysis-free survival in patients with diabetic nephropathy. The subjects were dichotomized based on the mean of the covariates: **a** 0.822 for miR-95-3p; **b** 0.938 for miR-631. *P* values refer to log-rank tests.

To investigate the effects of these microRNAs on the dialysis-free survival of DN patients, we performed a multivariate Cox regression analysis (Table 4). A higher miR-95-3p level decreased the risk of progression to ESRD and treatment with dialysis (*p* = 0.016, odds ratio (OR) = 0.305). However, a higher miR-631 level increased the risk of progression to ESRD and treatment with dialysis (*p* = 0.013, OR = 7.890). To further explore the effects of miR-95-3p and miR-631 on the dialysis-free survival of DN patients, we performed a Kaplan–Meier analysis (Fig. 4). The subjects were dichotomized based on the mean of the covariates (0.822 for miR-95-3p and 0.938 for miR-631). A high level of miR-95-3p decreased the risk of progression to ESRD (Fig. 4a, *p* = 0.002), while a high level of miR-631 increased the risk of progression to ESRD (Fig. 4b, *p* = 0.011).

**Table 4:** Multivariate Cox regression analysis for dialysis-free survival of patients with diabetic nephropathy.

Items	*β*	SE	Wald	*P*	OR	95% CI	
						Lower	Upper
miR-95-3p	−1.189	0.495	5.773	0.016*	0.305	0.116	0.803
miR-185-5p	−0.041	0.573	0.005	0.943	0.960	0.312	2.952
miR-1246	−0.738	0.575	1.645	0.200	0.478	0.155	1.477
miR-631	2.066	0.833	6.155	0.013*	7.890	1.543	40.344

*β* regression coefficient, *SE* standard error, *OR* odds ratio, *CI* confidence interval.

*p < 0.05.

## Discussion

In recent years, the incidence of diabetes has continued to increase, which has resulted in a heavy economic burden on society [28]. Diabetic patients with kidney disease could have DN or NDRD [9, 10]. Compared with NDRD, the treatment effect and prognosis of DN are worse than those of NDRD, and the pathological course is difficult to reverse [29]. The diagnostic gold standard for DN and NDRD is renal biopsy, which is an invasive procedure that has not been widely applied due to its high cost and high technical requirements [6, 30]. Therefore, the discovery of new noninvasive methods that can identify DN and NDRD has important clinical value. MicroRNAs have become biomarkers of many types of diseases due to their function and varied biological effects [17, 31, 32].

In the present study, microarray analysis was used to identify differentially expressed microRNAs in urinary sediment samples from DM, DN, and NDRD (IgAN and MN) patients. To expand the sample size for verification, many more urine samples were collected to confirm the candidate microRNAs and to construct diagnostic models in the confirmation cohort. We found that the levels of miR-95-3p, miR-185-5p, miR-1246, and miR-631 were all statistically significant in the DN group compared with the other groups. The diagnostic models we established had high diagnostic accuracy for distinguishing DN from the other groups (AUC = 0.863). The levels of miR-95-3p, miR-185-5p, miR-1246, and miR-631 were all statistically significant in the IgAN group and MN group compared with the DM group and DN group, while the levels of miR-185-5p and miR-631 were not statistically significant in the IgAN group and MN group. The diagnostic models we established had high diagnostic accuracy for distinguishing IgAN from the other groups (AUC = 0.859) and had moderate diagnostic accuracy for distinguishing MN from the other groups (AUC = 0.792).

Many additional urine samples were collected to externally examine the accuracy of the diagnostic models in the validation cohort. The Model DM, which is based on two microRNAs (miR-185-5p and miR-1246), exhibited high diagnostic accuracy (AUC = 0.928) in the validation cohort for distinguishing DM from the other groups. The Model DN, which is based on two microRNAs (miR-95-3p and miR-631), exhibited high diagnostic accuracy (AUC = 0.844) in the validation cohort for distinguishing DM from the other groups. Model IgAN, which is based on two microRNAs (miR-185-5p and miR-1246), demonstrated high diagnostic accuracy (AUC = 0.849) in the validation cohort for distinguishing IgAN from the other groups. However, Model MN, which is based on three microRNAs (miR-95-3p, miR-185-5p, and miR-1246), demonstrated moderate diagnostic accuracy (AUC = 0.761) in the validation cohort for distinguishing MN from the other groups.

We then explored the relationship between these microRNAs and disease severity and prognosis. We observed that the levels of miR-95-3p and miR-631 reflected the severity of DN. The Pearson correlation analysis revealed that the level of miR-95-3p was positively correlated with eGFR but was negatively correlated with the level of serum creatinine, classes of glomerular lesions, and scores of interstitial and vascular lesions. The level of miR-631 was positively correlated with proteinuria. Different severity levels of DN directly determine the judgment of the treatment effect and adjustment of the treatment plan. Therefore, the levels of miR-95-3p and miR-631 could play an important role in clinical decision-making in patients with DN. In addition, our multivariate Cox regression analysis and Kaplan–Meier analysis also demonstrated that low levels of miR-95-3p and high levels of miR-631 increased the risk of progression to ESRD. Therefore, the levels of miR-95-3p and miR-631 could play an important role in the prognostic prediction of DN.

Urine contains many disease biomarkers that reflect an individual’s health status and that are good indicators of kidney disease [33]. Recent studies have reported that many types of kidney diseases, such as primary IgAN, lupus nephritis, and minimal change nephropathy, could be detected by biomarkers in urinary sediment [34, 35]. Duan et al. found that the levels of miR-25-3p, miR-144-3p, and miR-486-5p were significantly higher in the IgAN group than in the normal control group [36]. Yan et al. found that urinary sediment could help with the differential diagnosis of lupus nephritis with endocapillary proliferative glomerulonephritis (EPGN) and IgAN with EPGN [35]. Compared with urine supernatant, urinary sediment was shown to be relatively less affected by humoral metabolic factors. Therefore, microRNAs in urinary sediment have the potential to serve as biomarkers for disease diagnosis and monitoring because they are relatively stable and are easily quantified. In addition, urinary sediment is obtained by a noninvasive method that can contribute to the early screening and monitoring of DN.

This study has a few limitations. First, our investigation did not refer to the mechanisms that cause alterations in microRNAs in patients with DN. Second, this was a single-center study, and it would be better if further multicenter studies and larger cohort studies are conducted for validation. Third, to further confirm the effect of microRNA levels on the prognosis of DN, it would be better to extend the follow-up time.

In conclusion, measurement of the levels of miR-95-3p, miR-185-5p, miR-1246, and miR-631 could be a useful and noninvasive tool for distinguishing patients with DM, DN, and NDRD (IgAN and MN). The levels of miR-95-3p and miR-631 can also reflect the severity and prognosis of DN.

## Supplementary information

supplementary materials
