# Supplementary material for: Urinary sediment microRNAs can be used as potential noninvasive biomarkers for diagnosis, reflecting the severity and prognosis of diabetic nephropathy
Source: Nutr Diabetes. 2021 Jun 30;11:24. doi: 10.1038/s41387-021-00166-z (PMC8245546; doi:10.1038/s41387-021-00166-z)
Supplement: Supplementary file 1 — supplementary materials [file 41387_2021_166_MOESM1_ESM.docx]

Figure S1. Global urinary sediment microRNA profiling among different groups of patients in a screening cohort.


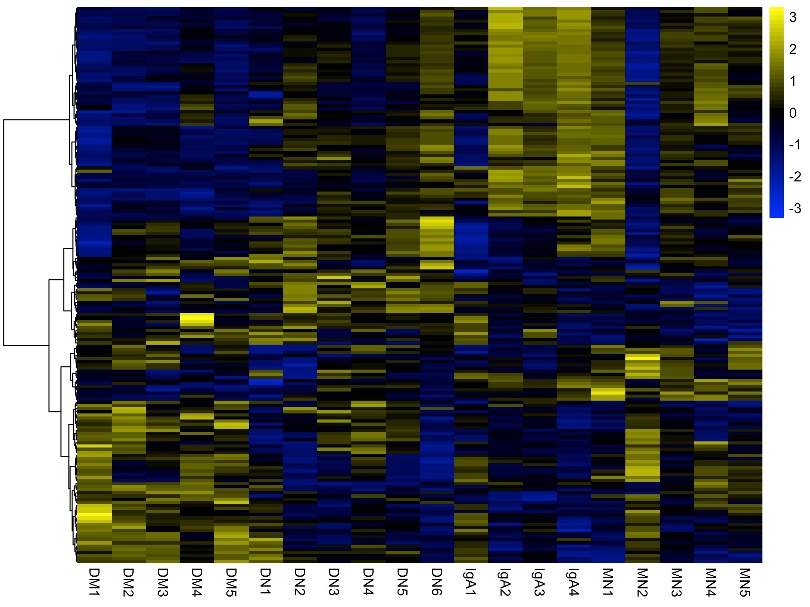


Abbreviations: DM, diabetes mellitus; DN, diabetic nephropathy; IgAN, IgA nephropathy; MN, membranous nephropathy.

Figure S2. Volcano plot of microRNAs in the DN group and DM group evaluated in the screening phase.


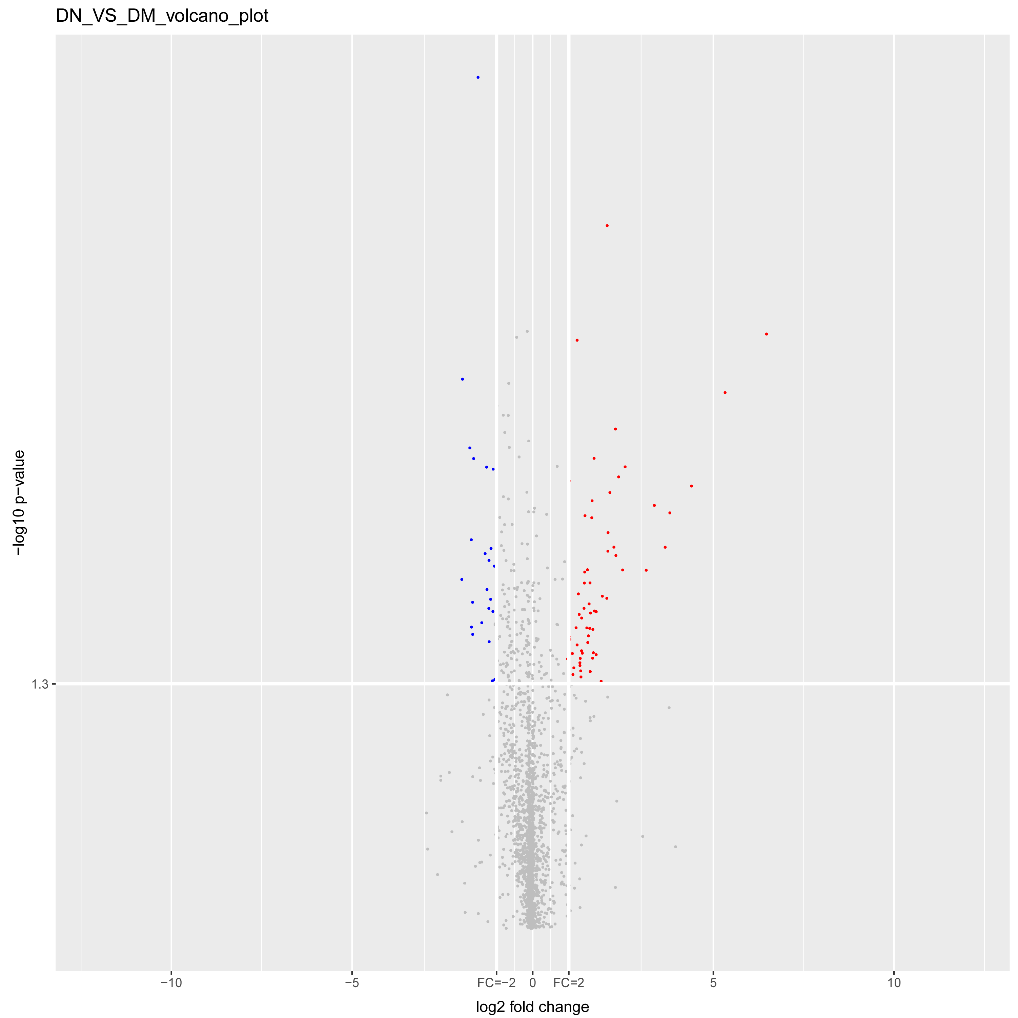


Figure S3. Volcano plot of microRNAs in the DN group and IgAN group evaluated in the screening phase.


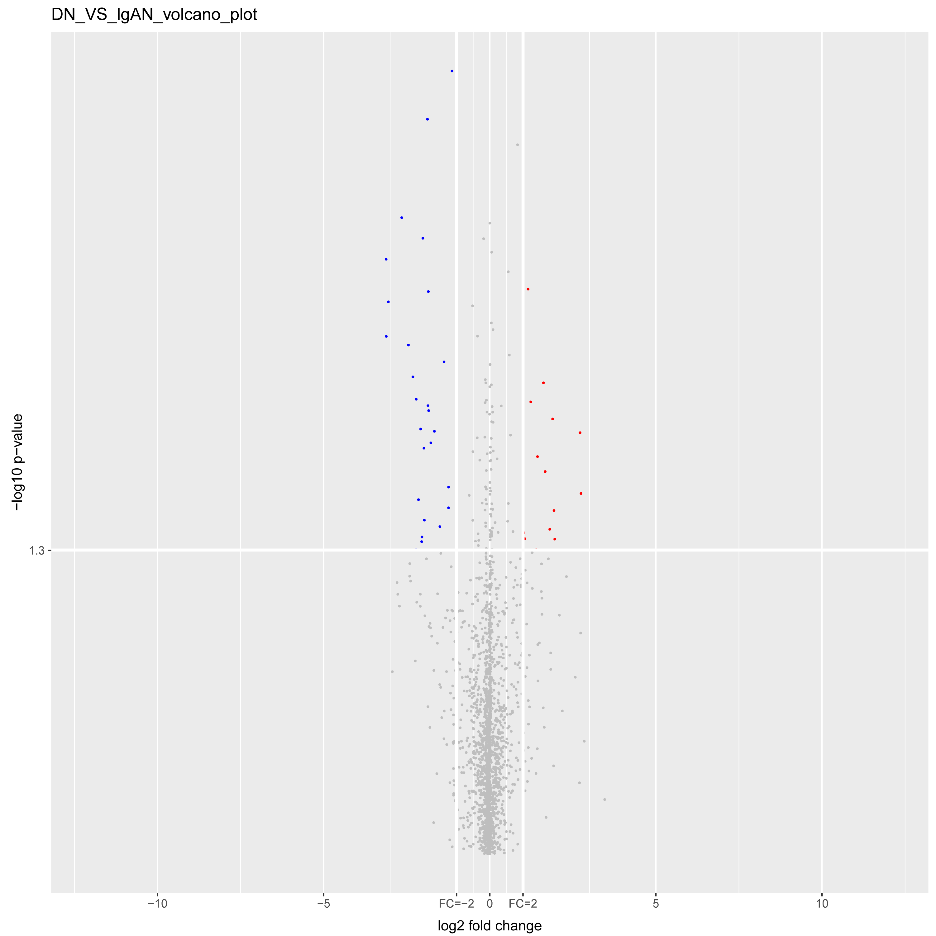


Figure S4. Volcano plot of microRNAs in the DN group and MN group evaluated in the screening phase.


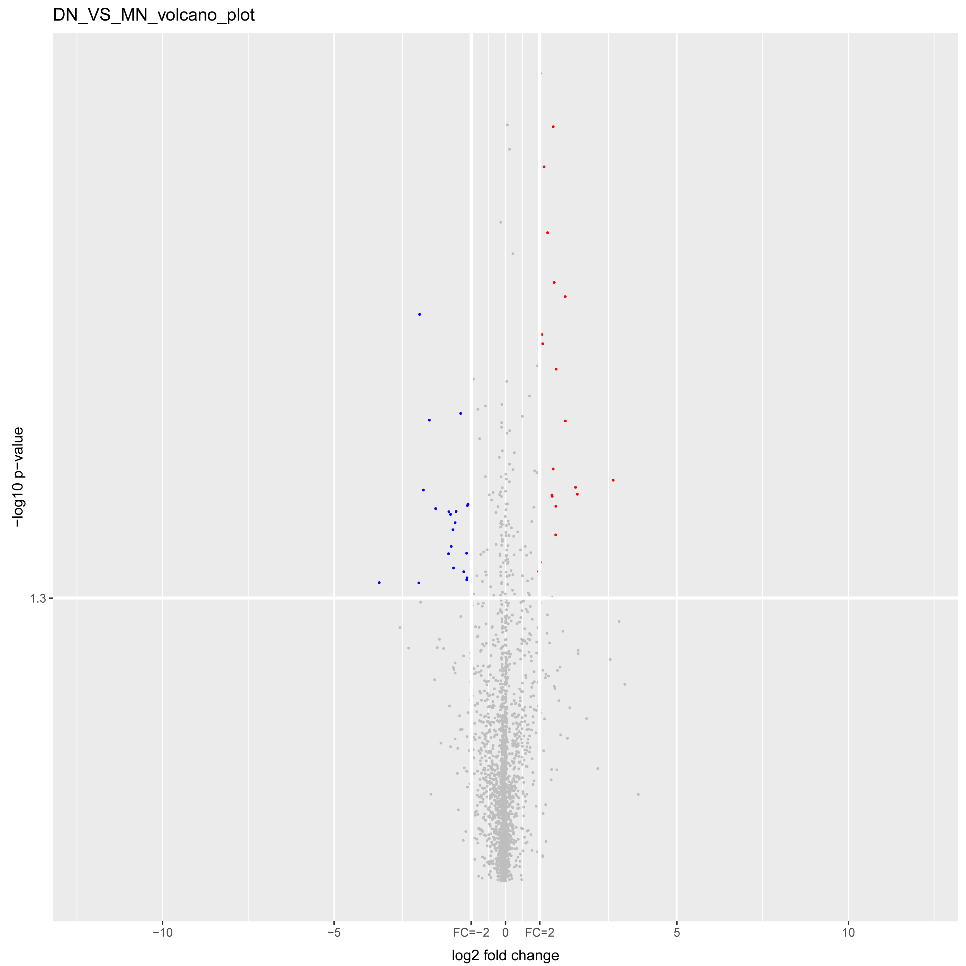


Figure S5. Volcano plot of microRNAs in the DM group and IgAN group evaluated in the screening phase.


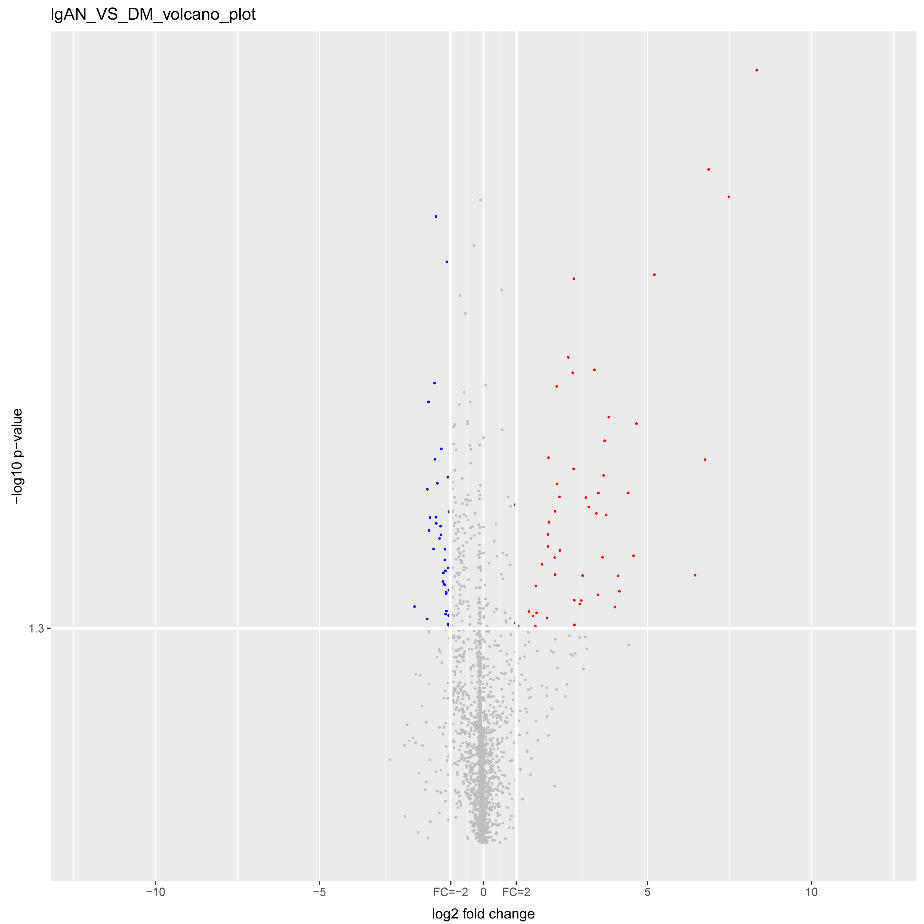


Figure S6. Volcano plot of microRNAs in the DM group and MN group evaluated in the screening phase.


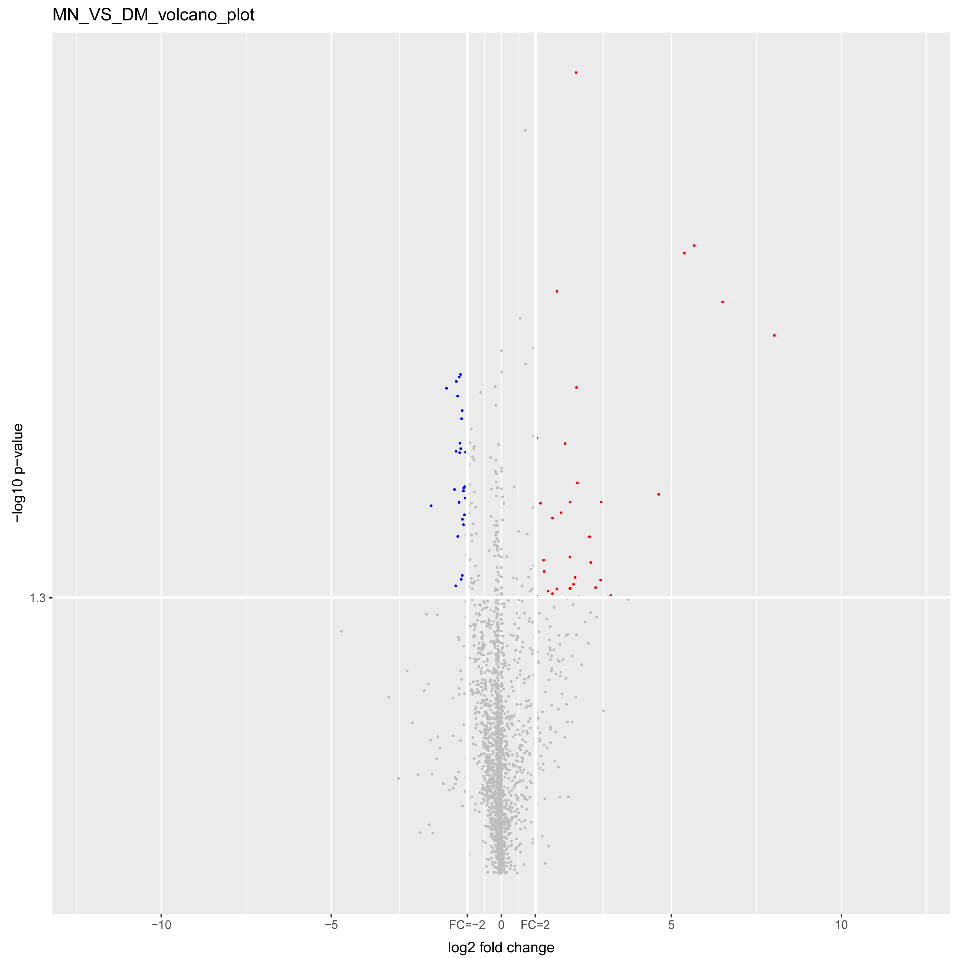


Figure S7. Volcano plot of microRNAs in the MN group and IgAN group evaluated in the screening phase.


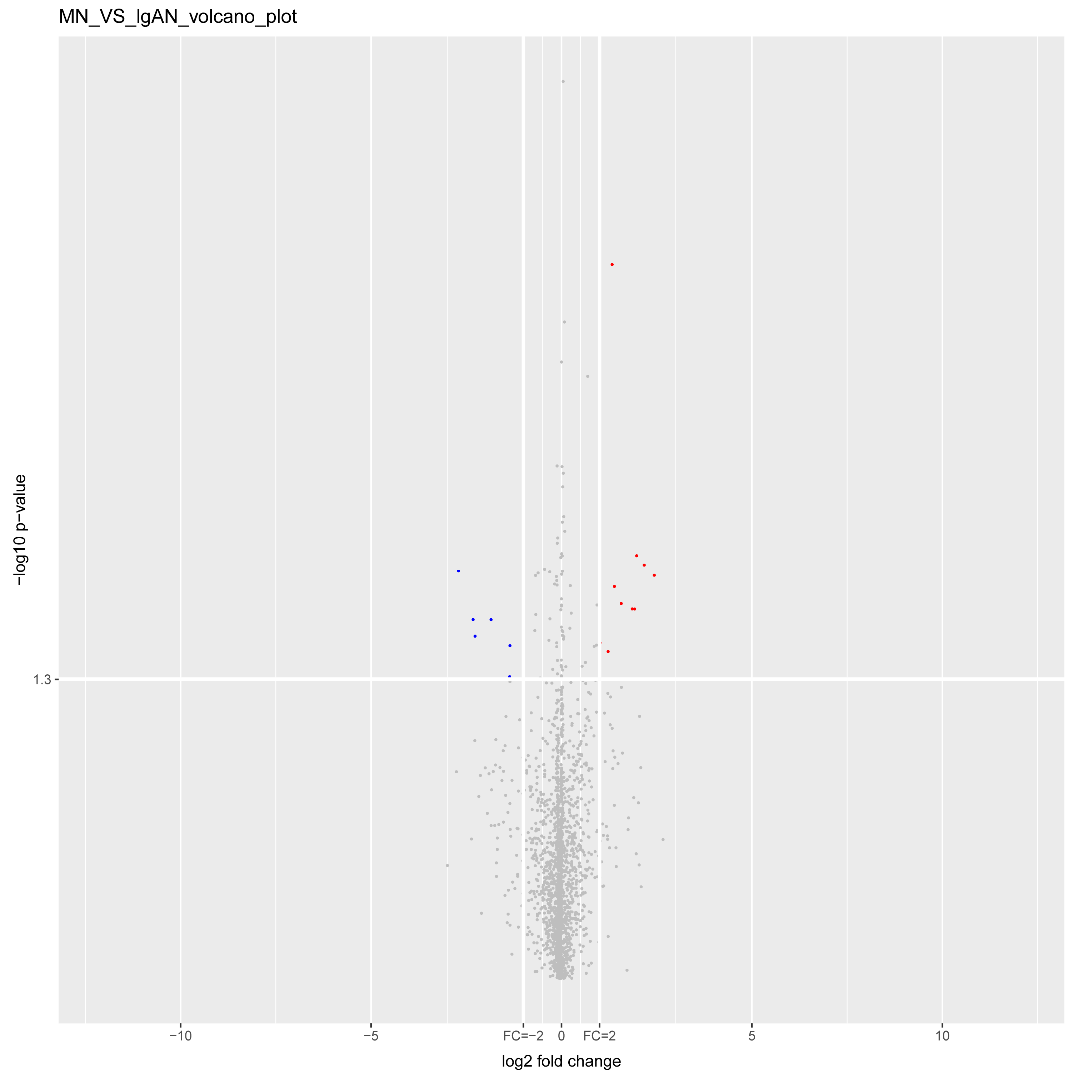


Table S1. Total numbers of microRNAs evaluated in the screening phase.

| Group name | Number of differentially expression genes |
| --- | --- |
| DN vs DM | 73 |
| DN vs IgAN | 39 |
| DN vs MN | 35 |
| DM vs IgAN | 90 |
| DM vs MN | 54 |
| MN vs IgAN | 16 |

Table S2. Baseline characteristics of subjects in the three cohorts.

| Characteristic | DM Group | DN Group | IgAN Group | MN Group |
| --- | --- | --- | --- | --- |
| The screening cohort | | | | |
| Age (years) | 52.40±9.79 | 58.33±13.16 | 59.25±13.84 | 52.80±7.69 |
| Male % | 40.00% | 50.00% | 50.00% | 60.00% |
| BMI (kg/m2) | 25.11±1.84 | 26.27±1.78 | 25.72±1.72 | 25.33±2.35 |
| SBP (mmHg) | 137.60±6.11 | 143.00±5.69 | 144.25±5.56 | 138.40±11.63 |
| DBP (mmHg) | 79.80±9.26 | 88.33±7.66 | 92.00±4.83 | 86.80±12.01 |
| DM duration (years) | 7.80±2.17 | 7.67±2.16 | 9.25±1.71 | 8.20±2.49 |
| FBG (mmol/l) | 7.54±0.96 | 7.20±0.84 | 6.78±0.44 | 7.42±0.90 |
| HbA1c (%) | 7.98±1.02 | 8.70±1.35 | 8.03±1.30 | 8.66±0.70 |
| Serum creatinine (umol/l) | 95.88±6.92 | 158.37±28.80 | 162.48±10.74 | 153.28±5.10 |
| The confirmation cohort | | | | |
| Age (years) | 56.98±10.41 | 53.87±9.61 | 49.59±12.25 | 57.48±9.91 |
| Male % | 52.38% | 55.56% | 50.00% | 52.38% |
| BMI (kg/m2) | 25.65±3.54 | 27.33±4.07 | 27.07±3.66 | 26.07±2.95 |
| SBP (mmHg) | 137.67±15.34 | 143.96±16.41 | 144.18±21.50 | 142.33±21.08 |
| DBP (mmHg) | 85.05±10.07 | 90.36±11.39 | 89.05±11.44 | 88.86±10.60 |
| DM duration (years) | 9.02±4.66 | 10.54±4.52 | 8.45±3.39 | 8.43±3.14 |
| FBG (mmol/l) | 8.90±4.53 | 6.99±2.96 | 6.90±2.43 | 6.91±2.48 |
| HbA1c (%) | 9.18±2.82 | 7.12±1.79 | 6.79±1.26 | 6.36±0.84 |
| Serum creatinine (umol/l) | 73.07±17.04 | 167.87±96.02 | 161.19±70.94 | 160.33±66.57 |
| The validation cohort | | | | |
| Age (years) | 55.53±8.35 | 52.98±7.81 | 52.00±8.01 | 56.50±8.76 |
| Male % | 53.49% | 56.10% | 52.00% | 54.17% |
| BMI (kg/m2) | 26.21±2.70 | 27.06±2.62 | 27.35±2.48 | 26.43±2.55 |
| SBP (mmHg) | 136.05±12.90 | 146.22±14.35 | 140.24±7.74 | 140.00±12.72 |
| DBP (mmHg) | 79.72±8.30 | 88.71±10.21 | 85.72±8.90 | 82.75±11.49 |
| DM duration (years) | 9.95±4.84 | 11.02±4.45 | 10.40±4.20 | 10.54±2.93 |
| FBG (mmol/l) | 9.04±3.78 | 7.28±2.68 | 7.26±2.80 | 7.98±2.39 |
| HbA1c (%) | 7.97±1.03 | 8.07±1.00 | 7.82±0.92 | 8.35±1.03 |
| Serum creatinine (umol/l) | 72.79±15.24 | 166.14±59.33 | 163.30±63.00 | 165.20±42.51 |
|  |  |  |  |  |

Abbreviations: DM, diabetes mellitus; DN, diabetic nephropathy; IgAN, IgA nephropathy; MN, membranous nephropathy; BMI, body mass index; SBP, systolic blood pressure; DBP, diastolic blood pressure; FBG, fasting blood-glucose; HbA1c, hemoglobin A1c.

Table S3. Detailed information regarding the ROC analysis of the constructive models in the confirmation cohort and validation cohort.

|  | AUC | 95% CI | | Optimal cutoff  value | Sensitivity | Specificity |
| --- | --- | --- | --- | --- | --- | --- |
|  |  | Lower limits | Upper limits |  |  |  |
| Confirmation cohort | | | | | | |
| Model DM | 0.995 | 0.986 | 1.000 | 0.440 | 0.976 | 0.989 |
| Model DN | 0.863 | 0.797 | 0.929 | 0.494 | 0.717 | 0.894 |
| Model IgAN | 0.859 | 0.782 | 0.937 | 0.137 | 0.909 | 0.713 |
| Model MN | 0.792 | 0.694 | 0.891 | 0.281 | 0.671 | 0.872 |
| Validation cohort | | | | | | |
| Model DM | 0.928 | 0.884 | 0.973 | 0.740 | 0.884 | 0.856 |
| Model DN | 0.844 | 0.772 | 0.916 | 0.253 | 0.878 | 0.713 |
| Model IgAN | 0.849 | 0.777 | 0.921 | 0.083 | 0.840 | 0.750 |
| Model MN | 0.761 | 0.660 | 0.862 | 0.108 | 0.833 | 0.691 |

Abbreviations: DM, diabetes mellitus (type 2); DN, diabetic nephropathy; IgAN, IgA nephropathy; MN, membranous nephropathy; ROC, receiver operating characteristic; AUC, the area under the ROC curve; CI, confidence interval.
